# Proinsulin is sensitive to reflect glucose intolerance

**DOI:** 10.1111/jdi.13106

**Published:** 2019-07-13

**Authors:** Akinobu Nakamura, Hideaki Miyoshi, Shigekazu Ukawa, Koshi Nakamura, Takafumi Nakagawa, Yasuo Terauchi, Akiko Tamakoshi, Tatsuya Atsumi

**Affiliations:** ^1^ Department of Rheumatology, Endocrinology and Nephrology Faculty of Medicine and Graduate School of Medicine Hokkaido University Graduate School of Medicine Sapporo Japan; ^2^ Division of Diabetes and Obesity Faculty of Medicine and Graduate School of Medicine Hokkaido University Graduate School of Medicine Sapporo Japan; ^3^ Department of Public Health Faculty of Medicine and Graduate School of Medicine Hokkaido University Graduate School of Medicine Sapporo Japan; ^4^ Research Unit of Advanced Interdisciplinary Care Science Osaka City University Graduate School of Human Life Science Osaka Japan; ^5^ The Hokkaido Centre for Family Medicine Sapporo Japan; ^6^ Department of Endocrinology and Metabolism Graduate School of Medicine Yokohama City University Yokohama Japan

**Keywords:** β‐Cell function, Epidemiology, Proinsulin

## Abstract

**Aims/Introduction:**

We investigated associations between glucose tolerance and β‐cell function using a series of estimation methods in a population‐based study.

**Materials and Methods:**

Data from the Dynamics of Lifestyle and Neighborhood Community on Health Study were analyzed. A total of 489 participants (263 women) were divided into three groups: normal glucose tolerance (NGT), prediabetes (PDM) and diabetes group. We estimated β‐cell function by the homeostasis model assessment of β‐cell function, proinsulin level (PI), C‐peptide index, proinsulin‐to‐C‐peptide ratio (PI/CPR) and proinsulin‐to‐insulin ratio. Because data on all five parameters of β‐cell function showed skewed distributions, the values of these parameters were normalized by natural logarithmic (ln) transformation. Next, the association between glucose tolerance and β‐cell function among participants without diabetes was examined. In this analysis, glucose tolerance was assessed based on glycated hemoglobin levels.

**Results:**

In the crude analysis, ln(PI) and ln(PI/CPR) were significantly higher in the diabetes group than those in the PDM and NGT groups, and these parameters were significantly higher in the PDM group than in the NGT group. Only ln(PI) in the PDM group was significantly higher compared with that in the NGT group after adjustment for age, sex and body mass index (ln[PI]: PDM group 2.38 pmol/L, 95% confidence interval 2.29–2.47 pmol/L; NGT group 2.17 pmol/L, 95% confidence interval 2.12–2.22 pmol/L; *P *<* *0.05). In addition, ln(PI) levels were significantly and positively correlated with glycated hemoglobin quartile in participants without diabetes.

**Conclusions:**

Our results showed that PI was the most sensitive to reflect glucose intolerance.

## Graphical abstract

Proinsulin is the most sensitive to reflect glucose intolerance.

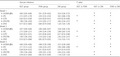

## Introduction

Previous studies have shown that deterioration of pancreatic β‐cell function or mass becomes apparent before a diagnosis of type 2 diabetes^1^, ^2^, ^3^, ^4^, ^5^, ^6^. Focusing on the natural history of type 2 diabetes progression, insulin secretion initially increases to compensate for peripheral insulin resistance. However, this increase in insulin secretion represents a relative shortage of insulin, and this impaired β‐cell function leads to the development of prediabetes and progression to frank type 2 diabetes^4^. Taken together, establishment of an evaluation method for estimating β‐cell function, which could show a strong association with glucose tolerance, would be expected.

Among several methods for estimating β‐cell function, assessment using parameters from fasting blood samples would be simple and clinically useful. However, it has not been clarified which parameters could show a strong association with glucose tolerance. The objective of the present population‐based study was to investigate associations between glucose tolerance and β‐cell function, as evaluated by five estimation methods, in a general Japanese population.

## Methods

### Study participants

In the present cross‐sectional study, we analyzed data from the Dynamics of Lifestyle and Neighborhood Community on Health Study (DOSANCO Health Study), as described previously^7^. In short, a total of 545 residents (300 women) in the town of Suttu, Hokkaido, Japan, aged 35–79 years, provided their basic information, including age, sex, medical history, anthropometric measurements and fasting blood samples. Of these 545 participants, those who had missing data on insulin levels (*n *=* *3) or were using antidiabetic agents (*n *=* *53) were excluded. The remaining 489 individuals (263 women) were considered as eligible study participants and included in the subsequent analyses. The study design was reviewed by the ethics board of Hokkaido University School of Medicine (15‐002 and 17‐015), and signed informed consent was obtained from all participants.

### Data collection

The weight and height of the participants were measured using a calibrated scale after they had removed their shoes and any heavy clothing. Body mass index (BMI) was calculated as weight in kilograms divided by height in meters squared. Venous blood samples were collected at rest in the morning after an overnight fast to measure levels of fasting plasma glucose (FPG), insulin, C‐peptide (CPR) and glycated hemoglobin (HbA1c). These parameters were measured using standard techniques. Proinsulin (PI) concentrations (pmol/L) were measured using a radioimmunoassay (Millipore Corporation Inc., Burlington MA, USA).

### Statistical analysis

Initially, glucose tolerance was categorized into the following three groups: normal glucose tolerance (NGT), prediabetes (PDM) and diabetes (DM). NGT was defined as FPG <110 mg/dL and HbA1c <5.7%, and PDM was defined as FPG 110–125 mg/dL or HbA1c 5.7–6.4%, or both^8^, ^9^. Participants were considered to have diabetes if they had a previous history of diabetes, FPG ≥126 mg/dL or HbA1c ≥6.5%^8^. β‐Cell function was estimated by homeostasis model assessment of β‐cell function (HOMA‐β%); PI; C‐peptide index (CPI), according to the formula 100 × fasting – CPR / FPG; ratio of PI‐to‐CPR (PI/CPR); and ratio of PI‐to‐insulin (PI/I)^10^, ^11^, ^12^.

Anthropometric and biochemical characteristics were crudely compared among the three groups regarding glucose tolerance, using one‐way analysis of variance, the Kruskal–Wallis test or the χ^2^‐test. Because data on all five parameters of β‐cell function showed skewed distributions, the values were normalized by natural logarithmic (ln) transformation. Comparisons of these log‐transformed parameters among the groups were assessed by analysis of covariance, followed by Tukey's honestly significant difference test for multiple post‐hoc comparisons. The model incorporated the following covariates: age (years, as a continuous variable), sex (male or female) and BMI (kg/m^2^, as a continuous variable).

Next, to explore a potential marker of early pancreatic β‐cell dysfunction, we examined the association between glucose tolerance and β‐cell function among participants without diabetes. In this analysis, glucose tolerance was assessed based on HbA1c levels. We compared anthropometric and biochemical characteristics in the participants grouped according to quartiles of HbA1c, using statistical methods the same as those used in the first analysis.

All tests were two‐sided, and *P *<* *0.05 was considered statistically significant. Statistical analysis was carried out using JMP 10 (SAS Institute Inc., Cary, NC, USA).

## Results

A total of 489 participants (263 women) were divided into three groups: NGT (*n *=* *328), PDM (*n *=* *113) and diabetes (*n *=* *48) groups. Anthropometric and biochemical characteristics of the participants are shown in Table 1. Age, proportion of women, BMI, waist circumference, and levels of insulin and CPR were positively associated with glucose intolerance. Table 2 shows β‐cell function, as evaluated by the five estimation methods, for each glucose tolerance group. In the crude analysis (model 1), ln(HOMA‐β%) was significantly lower in the diabetes group, but not in the PDM group, compared with the NGT group; ln(CPI) did not differ significantly among the three groups. Compared with the NGT group, ln(PI/I) was significantly higher in the diabetes group, but not in the PDM group. Of note, ln(PI) and ln(PI/CPR) were significantly higher in the diabetes group than in the PDM and NGT groups, and these parameters were significantly higher in the PDM group than in the NGT group. Similar results were observed for ln(PI) and ln(PI/CPR) after adjustment for age and sex (model 2). Only ln(PI) in the PDM group was significantly higher compared with that in the NGT group after adjustment for age, sex and BMI (model 3).

**Table 1:** Anthropometric and biochemical characteristics of 489 study participants

	Total participants	Glucose tolerance			*P*‐value
		NGT group	PDM group	DM group	
*n*	489	328	113	48	
Age (years)	58.0 ± 12.5	55.2 ± 12.7	63.7 ± 10.0	63.4 ± 9.9	<0.001
No. women (%)	263 (53.8)	186 (56.7)	62 (54.9)	15 (31.3)	0.004
BMI (kg/m^2^)	23.7 ± 3.6	23.3 ± 3.4	24.4 ± 4.0	24.4 ± 3.9	0.008
Waist circumference (cm)	81.6 ± 10.4	80.2 ± 9.9	83.7 ± 10.7	86.3 ± 11.5	<0.001
FPG (mg/dL)	93 (86–100)	90 (84–96)	99 (92–108)	128 (112–141)	<0.001
HbA1c (%)	5.4 (5.2–5.7)	5.3 (5.1–5.4)	5.8 (5.7–6.0)	6.5 (6.0–6.9)	<0.001
Ιnsulin (μU/mL)	4.3 (2.8–6.5)	4.0 (2.8–5.8)	5.2 (2.9–7.3)	6.0 (4.2–9.9)	<0.001
C‐peptide (ng/mL)	1.2 (0.9–1.7)	1.1 (0.9–1.5)	1.4 (1.0–1.9)	1.8 (1.2–2.5)	<0.001

Data are presented for the entire group and for participants grouped by their glucose tolerance. Values are expressed as mean ± standard deviation, median (interquartile range) or the number (%) of participants in that category. One‐way analysis of variance, Kruskal–Wallis test or χ^2^‐test were used to compare each parameter among the three glucose tolerance groups. BMI, body mass index; DM, diabetes; FPG, fasting plasma glucose; HbA1c, glycated hemoglobin; NGT, normal glucose tolerance; PDM, prediabetes.

**Table 2:** β‐Cell function evaluated by five estimation methods

	Glucose tolerance			*P* value		
	NGT group	PDM group	DM group	NGT vs PDM	NGT vs DM	PDM vs DM
Model 1						
ln (HOMA‐β%)	4.00 (3.93–4.06)	3.91 (3.79–4.02)	3.54 (3.36–3.72)		*	*
ln (PI)	2.13 (2.07–2.19)	2.43 (2.32–2.53)	3.02 (2.86–3.18)	*	*	*
ln (CPI)	0.26 (0.22–0.30)	0.34 (0.26–0.41)	0.29 (0.18–0.40)			
ln (PI/CPR)	1.98 (1.94–2.03)	2.10 (2.02–2.17)	2.46 (2.34–2.57)	*	*	*
ln (PI/I)	0.77 (0.72–0.83)	0.84 (0.74–0.93)	1.20 (1.05–1.34)		*	*
Model 2						
ln (HOMA‐β%)	3.97 (3.90–4.04)	3.96 (3.84–4.07)	3.58 (3.40–3.76)		*	*
ln (PI)	2.15 (2.09–2.21)	2.44 (2.34–2.54)	2.98 (2.82–3.14)	*	*	*
ln (CPI)	0.26 (0.22–0.31)	0.35 (0.28–0.43)	0.27 (0.16–0.38)			
ln (PI/CPR)	1.99 (1.94–2.03)	2.10 (2.02–2.18)	2.45 (2.33–2.57)	*	*	*
ln (PI/I)	0.79 (0.73–0.84)	0.82 (0.73–0.92)	1.16 (1.02–1.31)		*	*
Model 3						
ln (HOMA‐β%)	4.00 (3.93–4.06)	3.88 (3.78–3.99)	3.53 (3.38–3.69)		*	*
ln (PI)	2.17 (2.12–2.22)	2.38 (2.29–2.47)	2.94 (2.80–3.08)	*	*	*
ln (CPI)	0.28 (0.24–0.32)	0.31 (0.24–0.37)	0.24 (0.14–0.33)			
ln (PI/CPR)	1.99 (1.94–2.04)	2.09 (2.01–2.16)	2.44 (2.32–2.56)		*	*
ln (PI/I)	0.78 (0.72–0.83)	0.85 (0.75–0.94)	1.18 (1.03–1.32)		*	*

Data are presented for participants grouped according to their glucose tolerance. Values are normalized by natural logarithmic transformation and expressed as least squares means (95% confidence interval). Analysis of covariance and Tukey's honestly significant difference test were used to compare each parameter among the three groups. Model 1, crude; model 2, adjustment for age and sex; model 3, adjustment for age, sex and body mass index. *P <* *0.05. CPI, C‐peptide index; DM, diabetes; HOMA‐β%, homeostasis model assessment of β‐cell function; ln, natural logarithm; PI, proinsulin; PI/CPR, proinsulin‐to‐C‐peptide ratio; NGT, normal glucose tolerance; PDM, prediabetes; PI/I, proinsulin‐to‐insulin ratio.

As shown in Table 3, age, BMI, waist circumference, and levels of insulin and CPR were positively correlated with HbA1c quartile among the participants without diabetes. In the crude analysis (model 1), ln(PI) and ln(PI/CPR) were significantly and positively associated with HbA1c quartile, and the results were similar after adjustment for age and sex (model 2; Table 4). Only ln(PI) was significantly and positively correlated with HbA1c quartile in participants without diabetes, after adjustment for age, sex and BMI (model 3; Table 4).

**Table 3:** Anthropometric and biochemical characteristics of 441 participants without diabetes

	Total participants	HbA1c quartile				*P*‐value
		1st Quartile	2nd Quartile	3rd Quartile	4th Quartile	
*n*	441	151	100	81	109	
Age (years)	57.4 ± 12.6	52.2 ± 12.8	56.5 ± 12.5	59.4 ± 11.4	63.8 ± 9.9	<0.001
No. women (%)	248 (56.2)	78 (51.7)	57 (57.0)	52 (64.2)	61 (56.0)	0.334
BMI (kg/m^2^)	23.6 ± 3.6	22.7 ± 3.0	23.9 ± 3.7	24.0 ± 3.8	24.3 ± 3.8	0.002
Waist circumference (cm)	81.1 ± 10.2	78.5 ± 9.0	81.3 ± 10.8	82.3 ± 10.2	83.5 ± 10.6	0.001
FPG (mg/dL)	92 (85–98)	87 (83–94)	89 (85– 94)	95 (90–100)	98 (92–105)	<0.001
HbA1c (%)	5.4 (5.2–5.6)	5.1 (4.9–5.2)	5.4 (5.3–5.4)	5.5 (5.5–5.6)	5.9 (5.7–6.0)	<0.001
Ιnsulin (μU/mL)	4.1 (2.8–6.1)	3.8 (2.5–5.6)	4.0 (3.0–6.5)	4.2 (3.0–5.9)	5.2 (2.9–7.3)	0.012
C‐peptide (ng/mL)	1.1 (0.9–1.6)	1.0 (0.9–1.4)	1.1 (0.9–1.6)	1.1 (1.0–1.6)	1.4 (1.0–1.9)	0.002

Data are presented for the entire group and for participants grouped according to their glycated hemoglobin (HbA1c) levels. Values are expressed as mean ± standard deviation, median (interquartile range) or the number (%) of participants in that category. One‐way analysis of variance, Kruskal–Wallis test or χ^2^‐test were used to compare each parameter among the four groups. BMI, body mass index; FPG, fasting plasma glucose.

**Table 4:** β‐Cell function evaluated by five estimation methods

	HbA1c quartile			
	1st Quartile	2nd Quartile	3rd Quartile	4th Quartile
Model 1				
ln(HOMA‐β%)	4.00 (3.91–4.10)	4.06 (3.94–4.17)	3.93 (3.80–4.06)	3.89 (3.78–4.00)
ln(PI)	2.05 (1.97–2.14)	2.19 (2.08–2.29)	2.25 (2.13–2.36)*	2.41 (2.31–2.51)* ^,^ **
ln(CPI)	0.24 (0.18–0.30)	0.30 (0.22–0.37)	0.28 (0.19–0.36)	0.32 (0.25–0.39)
ln(PI/CPR)	1.95 (1.88–2.01)	2.00 (1.92–2.07)	2.03 (1.94–2.11)	2.10 (2.03–2.18)*
ln(PI/I)	0.76 (0.68–0.84)	0.76 (0.66–0.86)	0.77 (0.66–0.88)	0.86 (0.76–0.95)
Model 2				
ln(HOMA‐β%)	3.96 (3.86–4.06)	4.05 (3.93–4.17)	3.95 (3.81–4.08)	3.94 (3.83–4.06)
ln(PI)	2.06 (1.98–2.15)	2.20 (2.10–2.31)	2.28 (2.17–2.39)*	2.42 (2.32–2.52)* ^,^ **
ln(CPI)	0.23 (0.17–0.29)	0.31 (0.23–0.38)	0.30 (0.22–0.38)	0.35 (0.27–0.42)
ln(PI/CPR)	1.96 (1.89–2.02)	2.00 (1.93–2.08)	2.03 (1.94–2.11)	2.09 (2.02–2.17)*
ln(PI/I)	0.79 (0.71–0.88)	0.77 (0.67–0.87)	0.77 (0.66–0.88)	0.83 (0.73–0.93)
Model 3				
ln(HOMA‐β%)	4.04 (3.95–4.13)	4.02 (3.91–4.12)	3.89 (3.78–4.01)	3.87 (3.77–3.97)
ln(PI)	2.13 (2.05–2.20)	2.18 (2.09–2.27)	2.24 (2.14–2.34)	2.36 (2.27–2.45)* ^,^ **
ln(CPI)	0.28 (0.23–0.33)	0.29 (0.22–0.35)	0.27 (0.20–0.34)	0.30 (0.24–0.36)
ln(PI/CPR)	1.97 (1.90–2.03)	2.00 (1.92–2.08)	2.02 (1.94–2.11)	2.09 (2.01–2.16)
ln(PI/I)	0.76 (0.68–0.84)	0.78 (0.69–0.88)	0.79 (0.68–0.90)	0.86 (0.76–0.95)

Data are presented for participants grouped by glycated hemoglobin (HbA1c) level. Values are normalized by natural logarithmic transformation and expressed as least squares means (95% confidence interval). Analysis of covariance and Tukey's honestly significant difference test were used to compare each parameter among the four HbA1c quartiles. Model 1, crude; model 2, adjustment for age and sex; model 3, adjustment for age, sex and body mass index. *P <* *0.05 versus 1st Quartile, and **P <* *0.05 versus 2nd Quartile. CPI, C‐peptide index; HOMA‐β%, homeostasis model assessment of β‐cell function; ln, natural logarithm; PI, proinsulin; PI/CPR, proinsulin‐to‐C‐peptide ratio; PI/I, proinsulin‐to‐insulin ratio.

## Discussion

The present results showed that, of the five estimation methods, fasting PI was the strongest associated with glucose tolerance. Increased PI might be caused by an intrinsic defect in proinsulin processing or an increased secretory demand on β‐cells^13^. Indeed, consistent with the present results, fasting PI levels are significantly elevated not only in individuals with diabetes, but also in those with impaired fasting glucose and impaired glucose tolerance compared with those with NGT^14^, ^15^. Although PI/I and HOMA‐β% are known surrogate markers of β‐cell function^16^, we did not detect any significant differences in these markers between the NGT and PDM groups. It has been reported that PI/I might be affected by hepatic insulin clearance^12^, ^15^, and that HOMA‐β% could underestimate the magnitude of the β‐cell defect across declining glucose tolerance status, especially for impaired glucose tolerance^17^. CPI is mainly used as an index of endogenous insulin secretion to select the appropriate treatment for patients with type 2 diabetes 11. From the present results, however, it might not be useful for estimating β‐cell function in individuals with NGT, PDM or early type 2 diabetes. Therefore, fasting PI was the most sensitive to reflect glucose intolerance.

One limitation of the present study was that glucose tolerance was classified based on FPG and HbA1c levels. Prediabetes includes impaired fasting glucose and impaired glucose tolerance, which present with a different pathophysiology^18^. Thus, further studies are required to examine the usefulness of fasting PI as a marker to discriminate these conditions. Another limitation is that, because of its cross‐sectional design, the present study yielded no evidence on the time course of these parameters across various stages of glucose tolerance. Third, all participants in our study were Japanese, so whether our results are applicable to non‐Japanese populations remains unclear. Ethnic differences in the pathophysiological mechanisms of diabetes, including the degree of obesity and the insulin secretion capacity, have been documented between Japanese and Caucasians^19^, ^20^.

In conclusion, the present community‐based study showed that fasting PI was the strongest associated with glucose tolerance among the five estimation methods of β‐cell function. Considering that fasting PI levels were increased in participants with PDM, fasting PI is the most sensitive to reflect glucose intolerance.

## Disclosure

The authors declare no conflict of interest.
